# Lupus Anticoagulant-Hypoprothrombinemia Syndrome: A Duality Between Thrombosis and Hemorrhage

**DOI:** 10.7759/cureus.73149

**Published:** 2024-11-06

**Authors:** Laura Gago, Maria Helena Lourenço, Rita P Torres, Ana Filipa Mourão, Maria Manuela Costa, Jaime C Branco, Maria J Gonçalves

**Affiliations:** 1 Rheumatology, Egas Moniz Hospital, Unidade Local de Saúde de Lisboa Ocidental, Lisbon, PRT; 2 Comprehensive Health Research Center (CHRC), Nova Medical School, Lisbon, PRT; 3 Rheumatology, Unidade Local de Saúde da Cova da Beira, Covilhã, PRT

**Keywords:** blood coagulation factors, coagulation defect, lupus anticoagulant-hypoprothrombinemia syndrome, secondary antiphospholipid syndrome, systemic lupus erythematosus

## Abstract

Lupus anticoagulant-hypoprothrombinemia syndrome (LAHPS) is an uncommon but significant hematological disorder characterized by the presence of lupus anticoagulant (LA) and reduced levels of prothrombin (factor II). This syndrome presents a unique clinical paradox where patients may experience both a bleeding tendency due to hypoprothrombinemia and a prothrombotic state associated with LA. This syndrome should be suspected in the presence of increased coagulation times (prothrombin time and activated partial thromboplastin time) in association with the presence of LA. The dual risk between bleeding and thrombosis poses significant diagnostic and therapeutic challenges, particularly in deciding whether to use anticoagulation therapy, which could exacerbate bleeding, or withhold it, which could increase the risk of thrombosis. The authors report a case of a woman with LAHPS treated with rituximab. This case reinforces the importance of establishing guidelines for this disease.

## Introduction

Lupus anticoagulant-hypoprothrombinemia syndrome (LAHPS) is a rare disorder associated with the presence of lupus anticoagulant (LA) and hypoprothrombinemia, probably associated with antibodies against prothrombin (factor II) [1]. It can lead to higher bleeding risk, but several cases of thrombotic events have been described, mainly in patients with antiphospholipid syndrome (APS) [1-4], since the antiphospholipid antibody/prothrombin linkage increases the risk of thrombus formation. Very few cases are reported in the literature, mostly related to systemic lupus erythematosus (SLE), APS, and viral infections, and more commonly in pediatric age and young women [1]. This syndrome should be suspected in the presence of increased coagulation times (prothrombin time, PT, and activated partial thromboplastin time, aPTT) combined with the presence of LA [5]. Considering its rarity, treatment guidelines are not yet available. However, in cases associated with SLE, immunosuppression is recommended in the absence of spontaneous resolution of the clotting times, along with anticoagulation in case of thrombotic events [6]. The authors report a case of a middle-aged, Caucasian woman with a history of SLE and APS,referred to our center after experiencing multiple thrombotic events and prolonged coagulation times. Due to the bleeding risk associated with the elevated coagulation times, she had not received adequate anticoagulation treatment. Following a comprehensive diagnostic workup, she was diagnosed with LAHPS. Immunosuppression and carefully managed anticoagulation were initiated, resulting in a favorable clinical response and stabilization of hematologic parameters.

## Case presentation

A 58-year-old Caucasian woman, previously diagnosed with SLE with hematological (thrombocytopenia) and immunological involvement and secondary APS, was admitted to a Rheumatology Department for investigation of increased coagulation times. She had a history of episodes of significant metrorrhagia, five miscarriages after 12 weeks of gestation, thrombosis in left eye vessels (not possible to specify), ischemic stroke, and extensive acute myocardial infarction. In addition, she also had a history of severe left ventricular dysfunction (ejection fraction 34%). Myocardial perfusion scintigraphy showed apical and anteroapical necrosis without viability or ischemia.

She was only medicated with prednisolone (PDN) 20 mg/day for SLE. The cardiologists advised against the use of hydroxychloroquine (HCQ) due to the risk of a prolonged QT interval in a heart that is already experiencing ischemic heart insufficiency. Additionally, she has never been treated with other disease-modifying antirheumatic drugs. For her heart disease, she was taking ivabradine 10 mg/day, carvedilol 6.25 mg/day, furosemide 40 mg/day, sacubitril + valsartan 24+26 mg/day, and spironolactone 25 mg/day. Although she had a clear indication, neither treatment with anticoagulation was started nor an implantable cardioverter defibrillator was placed due to an increase in coagulation times.

Analytically, she presented high anti-double-stranded-DNA antibodies (54.3 international units, IU/mL; normal range <50 IU/mL), hypocomplementemia (C3: 61.4 mg/dL, normal range: 90-180; C4: 6.5 mg/dL, normal range: 10-40), triple antiphospholipid antibodies positivity in high-medium titles, thrombocytopenia (platelets 143,000/L), increased PT (18.8 seconds), prolonged aPTT (>120 seconds), and normal thrombin time (21 seconds). Factors II, V, VII, and X were reduced (Table 1). Mixing test of patient plasma with normal one resulted in no correction of coagulation times; levels of factors IX, XI, and XIII were corrected, but factor II was still low, suggesting the presence of inhibitors for this factor. Dosage of factors VIII and IX was performed by the chromogenic method, and their values were within the normal range. A thromboelastogram was completed, which was compatible with a state of hypercoagulability.

**Table 1:** Coagulation tests at admission and follow-up in the patient with lupus anticoagulant-hypoprothrombinemia syndrome PT: prothrombin time; aPTT: activated partial thromboplastin time; GPL: IgG phospholipid; GPI: glycoprotein I

Laboratory characteristics	Normal range	On admission	After 2 months	After 12 months
PT (seconds)	<14	18.8	20.4	17.4
aPTT (seconds)	23-38	>120	75.7	34.2
Factor II (%)	50-150	20	Not tested	Not tested
Lupus anticoagulant	-	Positive	Not tested	Not tested
Anticardiolipin antibody (GPL/mL)	-	-	Not tested	Not tested
IgG	<25	640	-	-
IgM	<25	0	-	-
Anti β2 GPI antibody (U/mL)	-	-	Not tested	Not tested
IgG	<25	867	-	-
IgM	<25	0	-	-

She was diagnosed with LAHPS and started immune-modulating therapy with azathioprine up to 150 mg/day (2 mg/kg/day). However, coagulation times did not improve, so treatment with intravenous rituximab (1,000 mg 15 days apart, six-month intervals) was started. Given the past occurrence of multiple thrombotic events, anticoagulation was started, initially with enoxaparin (60 mg twice a day) and subsequently switched to warfarin (variable dosage; 5 mg/day mostly), with no occurrence of new thrombotic or hemorrhagic events. Unfortunately, despite the stabilization of her clinical condition and laboratory parameters, the patient experienced several infectious complications requiring multiple hospital admissions, ultimately resulting in her death within this context.

## Discussion

The authors reported a case of LAHPS in a Caucasian, middle-aged woman with SLE and APS, with several thrombotic events. The first case of LAHPS was described in 1960 by Rapaport et al. [7], but it was not until 1982 that Bajaj et al. [5] identified the mechanism that causes this syndrome. The authors postulated that the development of autoantibodies targeting prothrombin (factor II), a vital protein in the coagulation cascade responsible for thrombin generation, can lead to accelerated clearance or functional inhibition of prothrombin, resulting in hypoprothrombinemia [5]. Inherited factor II deficiency is uncommon, whereas acquired deficiency is frequently found in severe liver disease, vitamin K deficiency, or vitamin K antagonist treatment. Factor II deficiency causes hypoprothrombinemia [8].

The presence of LA can induce venous and arterial thromboses and pregnancy morbidity, making it one of the main drivers of APS. Interestingly, unlike LAHPS, the presence of LA is usually associated with increased aPTT but not PT. While LA antibodies typically predispose individuals to thrombosis by interfering with anticoagulant pathways and promoting a prothrombotic state, the concurrent significant reduction in prothrombin levels tilts the balance [9]. Our patient only had a history of severe metrorrhagia in the past. This syndrome is more common in females and in pediatric age, which may be explained by the naturally faster hepatic clearance of the prothrombin/prothrombin antibody complex in children than in adults [10]. LAHPS could be associated with autoimmune diseases, most often described in patients with SLE, or associated with infectious diseases [1]. It can also be associated with neoplasms or drug abuse [3]. These triggers were not present in our patient.

LAHPS-reported cases have been associated with a wide spectrum of clinical manifestations, ranging from asymptomatic laboratory findings to life-threatening hemorrhages or thrombotic events [11-15]. The bleeding tendency in LAHPS is primarily due to hypoprothrombinemia and may present as mucosal bleeding, hematuria, or more severe bleeding events such as gastrointestinal or intracranial hemorrhage [16]. Paradoxically, despite the bleeding risk, patients may also develop thrombosis, such as the patient described, including deep vein thrombosis, pulmonary embolism, or arterial thrombosis [10,13-15]. These thrombotic events are described in patients with LAHPS and autoimmune disease but not with infections [17].

Diagnosing LAHPS requires a combination of clinical assessment, laboratory evaluations, and exclusion of other potential causes, such as bleeding disorders (clotting factors deficiency). It should be suspected when both PT and aPTT are prolonged, as well as positive LA. Normal thrombin time, fibrinogen, and platelet count are common findings [1,3,6,9,12,18]. Mixing tests are performed on a mixture of patient plasma and normal plasma and are indicated when the patient's coagulation test results are prolonged. It will differentiate between the presence of a factor deficiency and an inhibitor. Failure to correct the aPTT with the addition of normal plasma indicates the presence of an inhibitor in contrast with a factor deficiency [19]. In LAHPS, PT and aPTT fail to correct with normal plasma, indicating the presence of inhibitors. Testing for LA should be performed. In addition, coagulation factor activity should be measured. Factor II levels will be decreased in LAHPS. The activity of other coagulation factors may be artificially reduced, but repeating tests in increasing dilutions of plasma should elucidate the true level of these coagulation factors. In contrast, factor II activity will remain low at all dilutions [1].

Given the rarity of this syndrome, therapeutic guidelines are not yet available. Management of LAHPS is complex and requires a tailored approach based on the individual patient's risk of bleeding and thrombosis. The main goals are eradicating the inhibitor and controlling bleeding or thrombosis. In most cases described, PDN 1 mg/kg/day was used as the first therapeutic approach, with normalization of PT and aPTT values [1,3,8-14]. However, during corticosteroid tapering, PT and aPTT may increase again. It is therefore important to add a corticosteroid-sparing agent, with the most used being HCQ or azathioprine, with good efficacy [11,12]. Rituximab, an anti-CD20 monoclonal antibody, has been increasingly used in these cases after treatment failure with other drugs, with good results [1,14,15,20].

The decision to use anticoagulation in patients with LAHPS is particularly challenging. While anticoagulation may be necessary to prevent or treat thrombotic events, it must be carefully balanced against the risk of exacerbating bleeding. In most cases described with thrombotic events, anticoagulation was started initially with enoxaparin and later with the introduction of warfarin [8]. In case of severe bleeding, supportive care with plasma, vitamin K, prothrombin complex concentrate, or recombinant factor VII can be used, as well as red blood cells and platelet transfusions [1,8,14]. In our case, given the occurrence of multiple thrombotic events in the past, we initiated anticoagulation with enoxaparin in progressively higher doses until the therapeutic dose and subsequently switched to warfarin, with no occurrence of new thrombotic or hemorrhagic events.

In the present case, the therapeutic decision was made within a multidisciplinary team (which includes rheumatologists, immunohemotherapists, and clinical pathologists) to assess the risks and benefits of anticoagulation as well as the timing and dose on a regular basis.

## Conclusions

LAHPS is a rare but serious disorder that presents a complex clinical challenge. The paradox of simultaneous bleeding and thrombotic risks requires a nuanced approach to diagnosis and management. Clinicians must carefully balance the risks and benefits of anticoagulation and immunosuppression, tailored to the individual patient's clinical presentation and underlying disease. Early recognition and appropriate management are crucial to improving outcomes in this potentially life-threatening condition.
